# Supplementary material for: Routine CT Diagnostics Cause Dose-Dependent Gene Expression Changes in Peripheral Blood Cells
Source: Int J Mol Sci. 2025 Mar 29;26(7):3185. doi: 10.3390/ijms26073185 (PMC11989232; doi:10.3390/ijms26073185)
Supplement: Supplementary file 1 [file ijms-26-03185-s001.zip › ijms-3551601-supplementary.pdf]

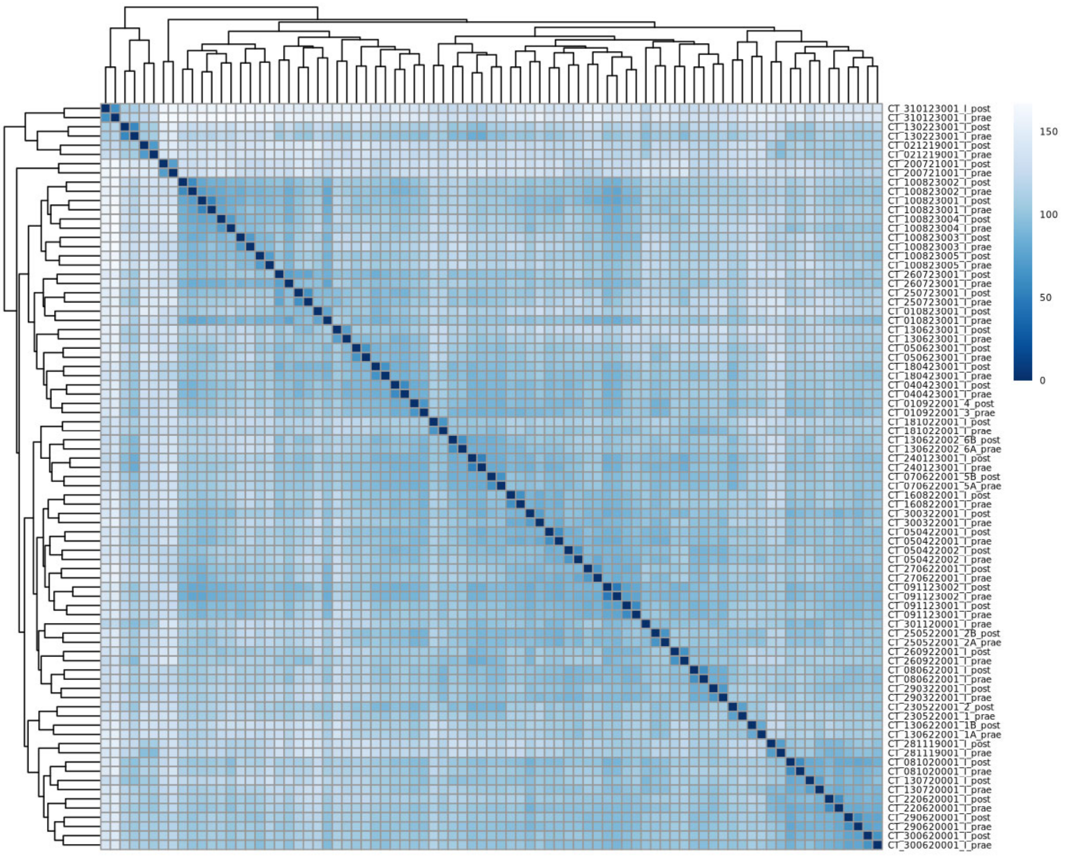

Supplementary Figure S1: Sample-to-Sample Distance Plot based on the Euclidean distance as calculated from the variance-stabilizing transformation of RNA-Seq count data from all RNA-Seq samples. The order of samples in combination with the dendrogram at the top or to the left of the heatmap denote the clusters of samples arising as a result of the hierarchical clustering process. High sample similarity within the heatmap is marked by dark blue as shown in the blue color gradient. Distinct clustering of the respective samples of each proband is shown without evidence for further characteristical attributes on gene expression signatures.

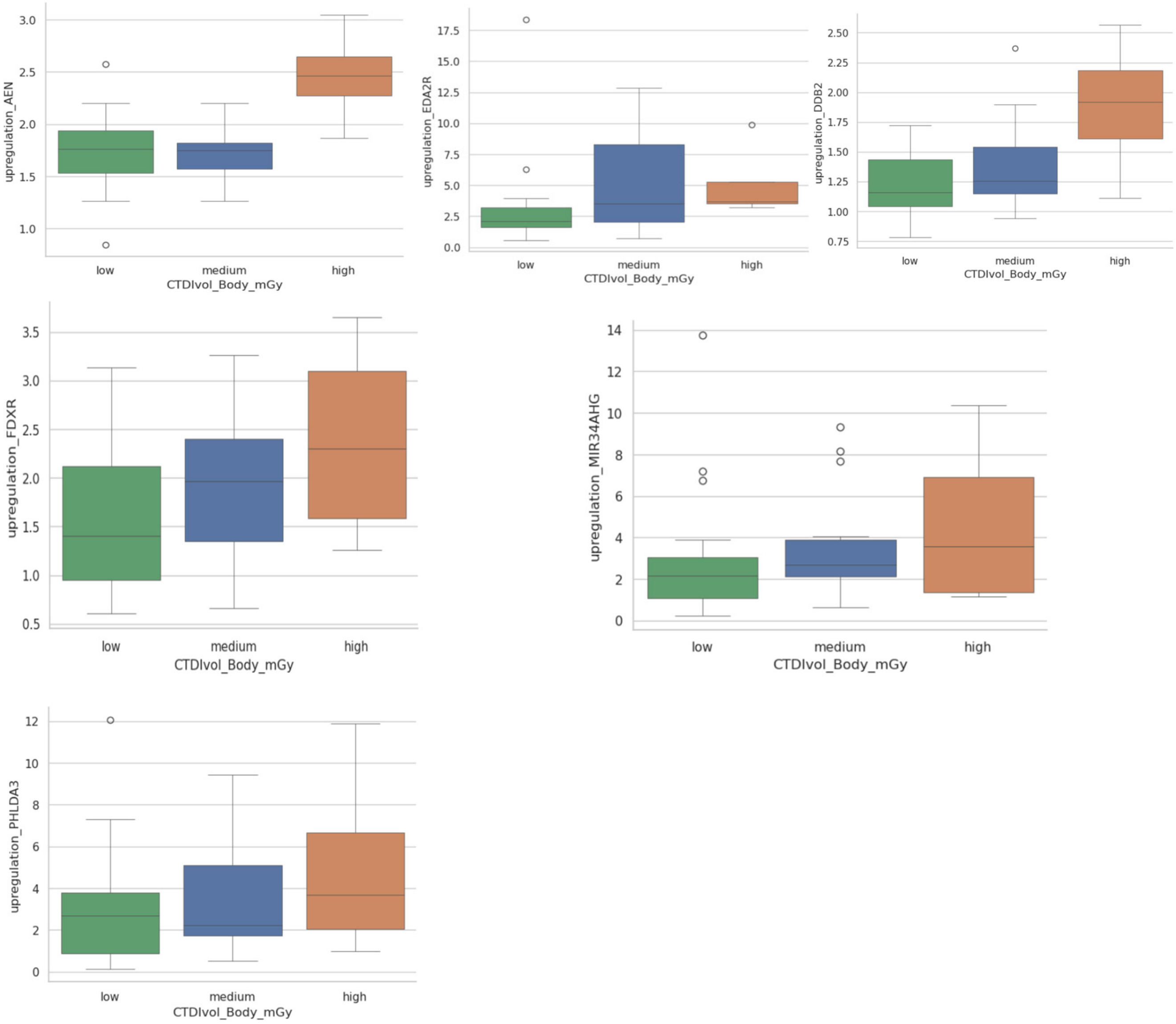

Supplementary Figure S2: Boxplots based on the ratio of normalized read counts per gene before and after exposure for each individual patient depicting patient specific upregulation of *AEN*, *DDB2*, *EDA2R*, *FDXR*, *MIR34AHG* and *PHLDA3*.
